# Changes in Medical Journals

**Published:** 1881-02

**Authors:** 


CHANGES IN MEDICAL JOURNALS.—The *American Medical Bi-Weekly*, which was formerly published at Louisville, Ky., and edited by E. S. Gaillard, M.D., was for a time discontinued on account of the failure of the editor's health. But Dr. Gaillard having recovered his health and established himself in New York city, has resumed the publication of his journal in that place.

The *Arkansas Medical Monthly*, heretofore published at Little Rock, Arkansas, is to be moved to Memphis, Tenn., and continued under a new name. J. J. Jones, M.D., will continue to be its chief editor.

We have received No. 1 of the *International Journal of Medicine and Surgery*, published at No. 1 Chambers street, New York, and edited by Drs. B. Newton, A. Rose, N. Senn, H. A. Bunker, and C. N. Ten Eyck.

---

*The Annals of Anatomy and Surgery*, is the slightly changed title of a medical periodical well known to many of our readers, as we have heretofore had occasion to reproduce some of its valuable and original work. It is now under the editorial management of Drs. L. S. Pilcher and Geo. R. Fowler, of Brooklyn, N. Y. We commend its fruitful pages most heartly to all our surgical and anatomical friends, as well as to all who are especially interested in the important field which it covers, and, for that matter, what medical is not.

